# The Structure of the NPC1L1 N-Terminal Domain in a Closed Conformation

**DOI:** 10.1371/journal.pone.0018722

**Published:** 2011-04-15

**Authors:** Hyock Joo Kwon, Maya Palnitkar, Johann Deisenhofer

**Affiliations:** 1 Department of Biochemistry, University of Texas Southwestern Medical Center, Dallas, Texas, United States of America; 2 Howard Hughes Medical Institute, University of Texas Southwestern Medical Center, Dallas, Texas, United States of America; University of Washington, United States of America

## Abstract

**Background:**

NPC1L1 is the molecular target of the cholesterol lowering drug Ezetimibe and mediates the intestinal absorption of cholesterol. Inhibition or deletion of NPC1L1 reduces intestinal cholesterol absorption, resulting in reduction of plasma cholesterol levels.

**Principal Findings:**

Here we present the 2.8 Å crystal structure of the N-terminal domain (NTD) of NPC1L1 in the absence of cholesterol. The structure, combined with biochemical data, reveals the mechanism of cholesterol selectivity of NPC1L1. Comparison to the cholesterol free and bound structures of NPC1(NTD) reveals that NPC1L1(NTD) is in a closed conformation and the sterol binding pocket is occluded from solvent.

**Conclusion:**

The structure of NPC1L1(NTD) reveals a degree of flexibility surrounding the entrance to the sterol binding pocket, suggesting a gating mechanism that relies on multiple movements around the entrance to the sterol binding pocket.

## Introduction

Cholesterol is an essential element of cell membranes, required for proper permeability and structural integrity. The proper regulation of cholesterols levels is critical for human health and individuals with elevated cholesterol levels have increased risks for coronary heart disease. Cholesterol also serves as an important signaling molecule, is the subject of feedback regulation, and serves as the precursor for steroid hormones. In humans, pathways including *de novo* synthesis, biliary excretion, and intestinal absorption maintain cholesterol homeostasis.

Dietary cholesterol is primarily absorbed in the intestine. After digestion in the lumen and hydrolysis of dietary lipids, cholesterol is solubilized in mixed micelles containing bile acid and phospholipids. This solubilization facilitates the movement of cholesterol from the bulk phase of the lumen to the surface of the enterocyte. Cholesterol absorption within enterocytes is mediated by Niemann-Pick C1 Like 1 (NPC1L1), a protein primarily expressed in the intestine [1] and the target of the cholesterol absorption inhibitor Ezetimibe [2]. NPC1L1 is a polytopic membrane protein that contains two conserved domains: the sterol sensing domain and the NPC1 domain. The NPC1 domain resides in the amino-terminal extracellular loop and is highly conserved in all NPC1 homologues.

Within the liver, cholesterol is secreted along with bile acids. In humans, NPC1L1 is also expressed in the liver. While the exact function of hepatic NPC1L1 is unknown, overexpression of NPC1L1 in livers of transgenic mice results in increased biliary reabsorption of cholesterol and increased plasma cholesterol [3]. A major source of cholesterol excretion in the liver is the ATP-binding cassette transporter ABCG5/G8 [4], which is also responsible for phytosterol excretion and NPC1L1 may play a role in fine tuning total cholesterol excretion in conjunction with ABCG5/G8.

Reduction or inhibition of NPC1L1 lowers intestinal cholesterol absorption by ∼70% emphasizing its key role in dietary cholesterol absorption. In transgenic mice, the effects of overexpression of NPC1L1 in the liver can be reversed by inhibition with Ezetimibe, reducing plasma cholesterol levels to normal. Thus, NPC1L1 is a proven target for lowering cholesterol from both dietary absorption and hepatic reabsorption.

Recently NPC1L1 has been shown to translocate between the plasma membrane and the endocytic recycling compartment in a cholesterol dependent manner. This translocation of NPC1L1 is blocked by Ezetimibe, and is specific for cholesterol over phytosterols [5], [6]. While phytosterol absorption has been shown to be NPC1L1 dependant [7], its absorption rate is lower than cholesterol and is inversely proportional to the size of the sterol, cholesterol > campesterol > β-sitosterol [8].

The mechanism through which NPC1L1 mediates cholesterol transport is unknown. NPC1L1 has high sequence homology (∼40%) to lysosomal NPC1, which mediates transport of LDL-derived cholesterol out of lysosomes for subsequent delivery to the endoplasmic reticulum and plasma membrane. NPC1 functions in tandem with NPC2, a soluble lysosomal protein, to move unesterified cholesterol [9]. Current models predict that NPC2 binds to unesterified cholesterol and delivers it to NPC1 for subsequent egress out of the lysosome. Cholesterol is virtually insoluble and NPC2 acts to solubilize cholesterol, allowing for transport in aqueous environments. While NPC2 works in conjunction with NPC1, NPC2 is not required for the function of NPC1L1 and mice lacking NPC2 absorb cholesterol normally [10]. While a protein partner for NPC1L1 has not been identified, it has been shown that cholesterol absorption by NPC1L1 in a cell culture model is dependent on the concentration of bile salt used to solubilize cholesterol in mixed micelles [11], [12]. Bile salt mixed micelles may serve an analogous function to NPC2, acting as a solubilizing agent to allow transport of cholesterol in aqueous environments and prevent precipitation/crystallization of cholesterol.

In previous work, we determined structures of NPC1(NTD) in both cholesterol bound and free forms. The structure of apo-NPC1(NTD) was nearly identical to that of cholesterol bound NPC1(NTD) and revealed a cholesterol binding pocket that was open to solvent. In the current study, we have determined the crystal structure of NPC1L1(NTD) to 2.8 Å resolution in a cholesterol free, closed form. The structure of NPC1L1(NTD), while sharing the same overall topology as NPC1(NTD), reveals a cholesterol binding pocket that is closed from solvent, suggesting a gating mechanism that relies on the movement of multiple elements around the cholesterol entrance. The cholesterol binding pocket is slightly larger than that of NPC1(NTD), suggesting broader sterol specificity. *In vitro* biochemical measurements of cholesterol binding and competition studies confirm this prediction.

## Results

A recombinant form of human NPC1L1(NTD) spanning residues 22–284 was produced in High-5 cells using a baculovirus vector. The soluble, secreted protein was purified from the culture media and the identity of the protein was confirmed by western blot analysis and N-terminal sequencing (data now shown). According to size exclusion chromatography, NPC1L1(NTD) exists as a monomer in solution (data not shown).

### Cholesterol binding specificity of NPC1L1(NTD)

**Figure 1:**
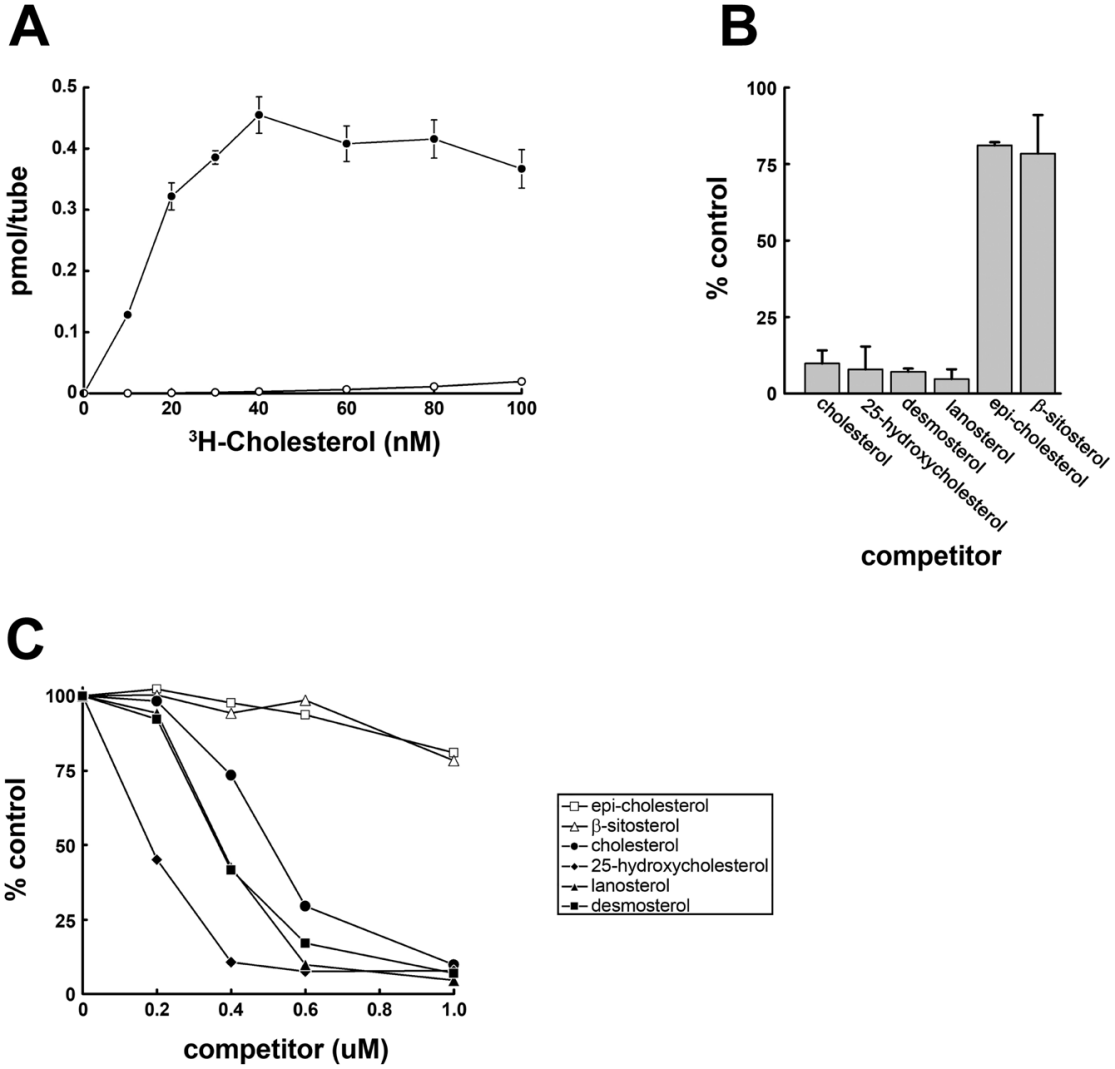
Sterol Binding and Specificity of NPC1L1(NTD). (A) Cholesterol binding. Each reaction, in a final volume of 100 µl, contained 0.5 pmol of purified HIS6-NPC1L1(NTD) and the indicated concentration of ^3^H-cholesterol in the absence (•) or presence (○) of 100 uM unlabeled cholesterol. Bound ^3^H-cholesterol was measured as described in *Materials and Methods*. Each data point represents the average of triplicate assays. (B) Competitive binding of ^3^H-cholesterol in the presence of unlabeled sterols. Each reaction, in a total volume of 100 µl, contained 0.5 pmol HIS6-NPC1L1(NTD), 10 nM ^3^H-cholesterol and 1 µM of the indicated unlabeled sterol. Each data point represents the average of triplicate assays and represents the amount of ^3^H-cholesterol bound relative to that in the control tube, which did not contain unlabeled sterol. (C) Competitive binding of ^3^H-cholesterol in the presence of unlabeled sterols. Each reaction, in a total volume of 100 µl, contained 0.5 pmol HIS6-NPC1L1(NTD), 10 nM ^3^H-cholesterol and varying concentrations of the indicated unlabeled sterol. Each data point represents the average of triplicate assays and represents the amount of ^3^H-cholesterol bound relative to that in the control tube, which did not contain unlabeled sterol.

To assay cholesterol binding to NPC1L1(NTD), we utilized an assay previously developed for monitoring cholesterol binding to SCAP [13]. ^3^H-cholesterol was incubated with His-tagged NPC1L1(NTD) and the protein was applied to a nickel-agarose column. Protein bound ^3^H-cholesterol was eluted and quantified by scintillation counting (Fig 1*A*). Binding of ^3^H-cholesterol was saturable with a K_d_∼12±4 nM. At saturation 0.5 pmol of NPC1L1(NTD) bound to ∼0.48±0.04 pmol of cholesterol suggesting a stoichiometry of one molecule of cholesterol for each NPC1L1(NTD) molecule.

We then carried out competitive binding studies to determine the specificity of various sterols toward NPC1L1(NTD). Various concentrations of unlabeled sterols were incubated with a fixed concentration of ^3^H-cholesterol and NPC1L1(NTD) and the ability of the unlabelled sterols to compete for binding was determined (Fig. 1*B,C*). Cholesterol, desmosterol, and lanosterol were equally effective in competing for cholesterol binding to NPC1L1(NTD), while 25-hydroxycholesterol (25HC) was slightly better. Epi-cholesterol and β-sitosterol were much less effective in competing for cholesterol binding to NPC1L1(NTD).

### Structure of NPC1L1(NTD)

**Figure 2:**
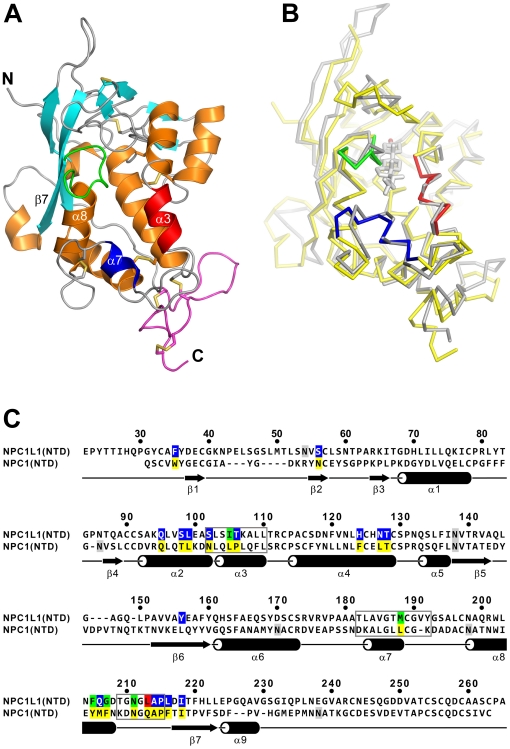
The Structure of NPC1L1(NTD). (A) NPC1L1(NTD) is represented as a ribbon diagram and the disulfide bonds are shown in yellow. Domain A is colored orange (α-helices), cyan (β-sheets), and gray (loops). α3 (red), α7 (blue), and the α8/β7 loop (green) surround the entrance of the cholesterol binding pocket. Domain B is colored magenta. (B) Superposition of NPC1L1(NTD) (yellow) and NPC1(NTD) (gray). Bound cholesterol in NPC1 is shown as a stick model. Regions around the entrance of the cholesterol binding pocket in NPC1L1 are colored red (α3), blue (α7), and green (α8/β7 loop). (C) Sequence alignment of NPC1L1(NTD) and NPC1(NTD). N-linked glycosylation sites are shaded gray. Residues lining the cholesterol binding pocket are shaded yellow in NPC1. In NPC1L1, residues within the interior of the closed cholesterol binding pocket are shaded blue, residues on the exterior of the closed cholesterol binding pocket are shaded green, and residues separating the interior from the exterior are shaded red. Regions around the entrance to the cholesterol binding pocket that change conformation are boxed. The secondary structure of NPC1L1(NTD) is shown below the sequence.

**Figure 3:**
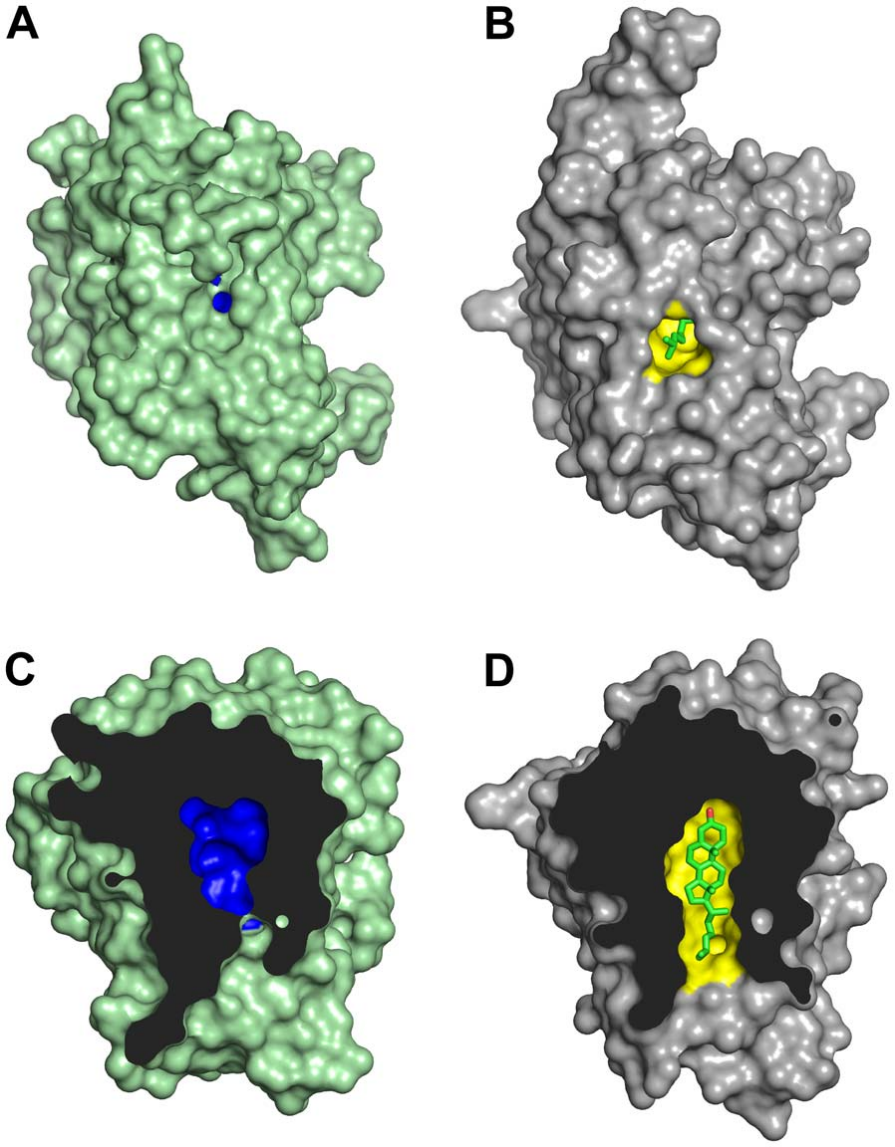
NPC1L1(NTD) in a closed conformation. (A) The surface of NPC1L1(NTD), colored green, reveals a cholesterol binding pocket (blue) that is closed to solvent. (B) The surface of NPC1(NTD), colored gray, shows the cholesterol binding pocket (yellow) exposed to solvent. The isooctyl side chain of cholesterol (stick model in green) is visible and solvent exposed. (C) Cutaway view of the cholesterol binding pocket of NPC1L1(NTD) in the closed conformation. (D) Cutaway view of the cholesterol binding pocket of NPC1(NTD) in an open conformation.

The structure of NPC1L1(NTD) in the absence of cholesterol was determined by molecular replacement and refined to 2.83Å (Table 1). The model spans residues 22–265. Residues 266–284 were poorly ordered and were not placed in the electron density. NPC1L1(NTD) is mainly helical, composed of nine α-helices, flanked by a mixed three strand β-sheet (Fig 2*A*). Electron density is present for N-acetyl glucosamine attached to ASN55 and ASN138. The structure can be divided into 2 domains, residues 22–242 (domain A), and residues 243–265 (domain B). Nine conserved disulfide bonds constrain the structure of NPC1L1(NTD), with Domain A containing six and Domain B containing two disulfide bonds. An interdomain disulfide (CYS113:CYS254) provides a second linkage between Domain A and B. A large central cavity, formed by helices α2,α3,α4,α7, and α8 is visible within NPC1L1(NTD) (Fig 3*A,C*). The cavity, while large enough to accommodate the tetracyclic ring of cholesterol, narrows as it reaches the surface, closing to a width of ∼2.3Å.

**Table 1:** Data collection and refinement statistics.

Space Group	C222_1_
a, Å	73.235
b, Å	107.133
c, Å	68.718
Resolution, Å (final shell)	50-2.83 (2.90-2.83)
Reflections	
Total	25279
Unique	6148
Completeness, %	92.3 (83.0)
R_sym_, %	9.4 (25.7)
Model Refinement Statistics	
Resolution, Å (final shell)	30-2.83 (2.91-2.83)
R_work_, %	22.6 (25.1)
R_free_, %	29.0 (42.1)
Rms deviations from target values	
Bond lengths, Å	0.006
Bond angles, °	1.027

### Comparison of NPC1L1 and NPC1

**Figure 4:**
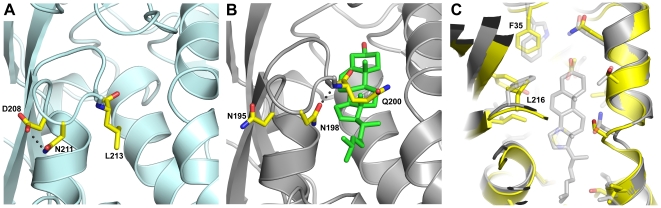
Structural differences between NPC1L1 and NPC1. (A) In the closed form of NPC1L1(NTD), ASN211 forms a hydrogen bond with ASP208, allowing LEU213 to rotate toward α7. (B) In the cholesterol bound form of NPC1(NTD), ASN198 forms a hydrogen bond with the main chain amine of GLN200, which is rotated away from α7. (C) Superposition of NPC1L1(NTD) (yellow) and NPC1(NTD) (gray) within the cholesterol binding pocket. The smaller residues of NPC1L1, PHE35 and LEU216, expand the cholesterol binding pocket, accommodating sterols such as lanosterol.

The structure of NPC1L1(NTD) is nearly identical to NPC1(NTD) with a rmsd across equivalent Cα atoms of 1.6Å (Fig. 2*B*). Helices 4, 6, and 8 are most similar between the proteins, with a rmsd across Cα atoms of 0.7Å and are spatially constrained by a disulfide bond between CYS116 and CYS172. Differences occur between the two proteins in regions around the entrance of the cholesterol binding pocket (Fig 2*B*, 3). The largest differences occurring in α3, α7, and the α8/β7 loop. Rotation of α3 and α7 in NPC1L1(NTD) relative to NPC1(NTD) results in a narrowing of the entrance to the sterol binding pocket. The most significant changes are in the α8/β7 loop, where LEU213 (GLN200 in NPC1) is rotated toward α7 displacing ASN211 (ASN198 in NPC1) which forms a hydrogen bond with ASP208 (Fig 4*A*). In NPC1(NTD), ASN198 forms a hydrogen bond with the main chain amine of GLN200, resulting in a shift of the α8/β7 loop and GLN200 away from α7 (Fig 4*B*). These differences result in a larger entrance to the sterol binding pocket in NPC1(NTD), with a width of ∼4.8Å.

## Discussion

Based upon the high sequence conservation (∼33% identity), it was expected that the overall fold of NPC1L1(NTD) would be similar to NPC1(NTD). The conformation of apo-NPC1L1(NTD) observed in the present work, however, reveals a closed conformation. Previously, we observed that apo-NPC1(NTD) was in an identical conformation as the cholesterol bound form and the cholesterol binding pocket was open to solvent [9]. As the cholesterol bound and unbound forms of NPC1(NTD) crystallized in the same spacegroup, the conformation of apo-NPC1(NTD) observed in our previous study may have been the result of crystal packing. Comparison of apo-NPC1L1(NTD) to the sterol bound forms of NPC1(NTD) suggests a gating model where multiple movements around the entrance to the sterol binding pocket are required to expand the entrance and allow entry of cholesterol.

The structure of NPC1L1(NTD) and the biochemical data presented here are consistent with *in vitro* cell culture assays demonstrating a high degree of specificity for cholesterol over other sterols. The structure of NPC1L1(NTD) reveals a slightly larger binding pocket than is present in NPC1(NTD) allowing for broader sterol specificity than NPC1 [14], [15], in particular sterols with substitutions at C4 (Fig. 4*C*). Residues near the C4 position are smaller in NPC1L1 (PHE35 and LEU216) compared to NPC1 (TRP27 and PHE203), increasing the size of the sterol binding pocket and accommodating C4 substituted sterols such as lanosterol. The contribution of NPC1L1 to the absorption of C4 substituted sterols has not been defined and further experiments will be required to determine what role it may have in the absorption of this class of sterols. While the residues near the C4 position are smaller in NPC1L1 compared to NPC1, residues around the isooctyl sidechain are roughly equivalent. The addition of an ethyl group at C24 on β-sitosterol would result in an unfavorable steric clash and we were unable to observe significant competitive binding of β-sitosterol to NPC1L1(NTD).

Ge *et. al.* [5] reported that cholesterol specifically promotes internalization of NPC1L1, whereas β-sitosterol and other sterols with substitutions at C24 had little effect on internalization of NPC1L1. This internalization was blocked by Ezetimibe, which has been shown to require the 2^nd^ extracellular domain of NPC1L1 for high affinity binding [16]. This result is surprising, given *in vivo* work in mice showing that NPC1L1 mediates phytosterol absorption [7]. Further experiments will be needed to address the mechanism of cholesterol transport by NPC1L1 and the functions of the different domains in cholesterol binding, transport and internalization.

## Materials and Methods

### Materials and Plasmids

^3^H-cholesterol was obtained from American Radiolabeled Chemicals; all other sterols were from Steraloids.

We generated a recombinant baculovirus encoding the N-terminal domain of human NPC1L1 by amplifying residues 22–284 of human NPC1L1 by PCR. The PCR fragment was digested with Sfo1 and EcoR1 and ligated into a modified version of pFastBacHTa (Invitrogen) that contains the honeybee mellitin signal sequence preceding a HIS6-tag [9].

### Sterol Binding

Each reaction contained, in a final volume of 100 µl of Buffer A (25 mM Tris pH 7.5, 150 mM NaCl) containing 0.0005% (v/v) NP-40), varying concentrations of the indicated sterols and 0.5 pmol of NPC1L1(NTD). After incubation for 24 hr at 4°C, each assay mixture was loaded onto a 2-ml column packed with 0.2 ml of Ni-NTA-agarose beads that had been pre-equilibrated with buffer A containing 0.0015% (v/v) NP-40 and then washed with 10 ml of buffer A containing 0.0015% (v/v) NP-40. Protein-bound ^3^H-cholesterol was eluted with 1.2 ml of buffer A containing 0.0015% (v/v) NP-40 and 250 mM imidazole and quantified by scintillation counting.

### Protein Expression and Purification

Sf9 cells infected with NPC1L1(NTD) baculovirus [17] were used to infect High-5 cells (Invitrogen) at 1×10^6^ cells/ml in Excel-405 medium. After incubation for 96 hr at 27°C, cells were pelleted by centrifugation, and the medium was concentrated by tangential flow filtration. NPC1L1(NTD) was purified from the concentrated medium by Ni-NTA chromatography. NPC1L1(NTD) was further purified by ion exchange chromatography followed by size exclusion chromatography. For crystallization the HIS6-tag was removed by tobacco etch virus protease cleavage prior to ion exchange chromatography.

### Crystallization and Structure Determination

Initial crystals were obtained using the Fluidigm TOPAZ system. Crystals of NPC1L1(NTD) were produced by mixing protein at 15 mg/ml with an equal volume of reservoir solution containing 0.9 M Li_2_SO_4_ and 100 mM MES pH 6.5. Crystals were transferred stepwise into reservoir solution containing 25% (v/v) glycerol and flash-frozen in a −160°C nitrogen stream. The crystals belong to space group C222_1_. Diffraction data were collected at the Advanced Photon Source (Argonne, IL, USA) beam line 19-ID and processed with HKL2000 [18] and the CCP4 suite [19]. The structure was determined by molecular replacement using the program PHASER [20]. The apo structure of NPC1(NTD) was used as the search model (Protein Data Bank ID code 3GKH). The model was built with the program COOT [21]. Initial refinement was performed with CNS [22], and the final cycles of refinement were performed with REFMAC [23]. Figures were generated with PYMOL (http://www.pymol.org).

### Accession Codes

The atomic coordinates have been deposited in the Protein Data Bank, www.pdb.org (PDB ID code 3QNT).
